# Launching the Tree of Life Gateway

**DOI:** 10.12688/wellcomeopenres.16913.1

**Published:** 2021-05-21

**Authors:** Jonathan Threlfall, Mark Blaxter

**Affiliations:** 1 Tree of Life, Wellcome Sanger Institute, Hinxton, Cambridge, UK

**Keywords:** Genome sequence, chromosomal, biodiversity, ecology, genomics

## Abstract

The Tree of Life Gateway uses Genome Note publications to announce the completion of genomes assembled by the Tree of Life programme, based at the Wellcome Sanger Institute and involving numerous partner organisations and institutes. Tree of Life participates in the Darwin Tree of Life Project, which aims to sequence the genomes of all 70,000+ eukaryotic species in the Atlantic archipelago of Britain and Ireland, the Aquatic Symbiosis Genomics Project, which will sequence 1000 species involved in 500 symbioses between eukaryotic hosts and their microbial 'cobionts', and other initiatives, such as the Vertebrate Genome Project. These Genome Notes report the origins of ethically sourced samples used for sequencing, give the methods used to generate the sequence and use statistics and interactive figures to demonstrate the quality of the genome sequences. In addition to describing the production of these sequences, each Genome Note gives citeable credit to those who participated in producing the genome assembly and announces the availability of the data for reuse by all. It is through the use and reuse of this openly and publicly released data that we hope effective and lasting solutions to the ongoing biodiversity crisis can be found.

The 300-year old Linnaean project aims to provide a catalogue of the names of all of the species on our planet, a catalogue of the books of life. The much younger Earth BioGenome Project ( Lewin *et al.*, 2018) and related efforts aim to take the Linnean project a step further and to make evident the content of the books of life: the complete genome sequences of all species. These sequences promise to revolutionise understanding of the natural world, and to be the foundation of future research in biological science, biotechnology, and data-driven conservation. Importantly, the community intends that these genomes will be generated from legally and ethically sourced specimens, and will be of the highest quality (chromosomally complete), so they can form a lasting resource for all humanity.

This Gateway collects Genome Note publications generated by the Tree of Life programme at the Wellcome Sanger Institute and our collaborators as part of our contributions to the overarching goals of the Earth BioGenome Project. Each Genome Note reports in the published and community peer-reviewed record a statement for each completed genome that evidences its origin (location, ethical and legal sampling, etc.), summarises and demonstrates its quality, and announces its availability for open reuse. These Genome Notes also serve to give clear, citable credit to the people who worked to make the genome assemblies possible, from field collectors and taxonomists, to database curators. In publishing them openly we also make clear that the data are available for reuse by all, without embargo. This mode of publication of genome sequences has become relatively common in virology and bacteriology, but for eukaryotic species there has been a tradition of publication of each genome in an analytic manuscript. This publication model is less and less tenable in the face of the increasing rate of sequence generation, and also results in significant delay between genome assembly and public data availability. We believe that publication of Genome Notes will deliver to the goals of findability, accessibility, interoperability and reuse (the FAIR principles) and will maximise the impact of these data.

Tree of Life participates in a number of national and international collaborations that include museums, botanical gardens and other biodiversity organisations, and institutes focussed on data analysis and databasing. The Darwin Tree of Life project aims to sequence the genome of every eukaryotic species of life in the Atlantic archipelago of Britain and Ireland, currently estimated to include over 70,000 taxa. To make this a reality, the Darwin partnership will initially sample at least one representative species from each taxonomic Family found in Britain and Ireland (about 4200 species) before proceeding to complete the remaining species. Tree of Life also leads the Aquatic Symbiosis Genomics project, which will sequence the genomes of 1000 species involved in 500 symbioses between eukaryotic macrobionts (“hosts”) and their microbial cobionts. Other genome sequences from Tree of Life, such as those generated as part of the Vertebrate Genomes Project, will also be deposited as Genome Notes and linked in this Gateway

The genomic data that are being generated by Tree of Life will have utility across many disciplines and fields. They will drive understanding of the different communities of species that live in Britain and Ireland in unprecedented depth, and, given that most British and Irish species are also found in mainland Europe, assist in the development of the European Reference Genome Atlas, which aims to sequence all eukaryotes in Europe. The genomes will be used to decipher the evolutionary histories of these species, and reveal the dynamic processes of evolution at the genomic scale. They act as foundational references that can be used to inform strategies to conserve species whose populations are at risk. The projects will also develop and refine wet lab and bioinformatic technologies to assure successful DNA extraction, sequence assembly and genome curation, and will share these openly to promote the global goal of sequencing all life.

In the face of what has been called the sixth great extinction, and threats to biodiversity and the ecosystem services that diverse species provide to sustain human society ( Hooper *et al.*, 2012), it is vital that biodiversity genome data and methods are made openly available for all. We hope that the initiation of the Genome Note mode of public data release will promote the data commons required to build effective and lasting responses and remedies for the ongoing biodiversity crisis.

## Data availability

No data is associated with this article.

## References

[ref-1] Hooper DU Carol Adair E Cardinale BJ: A Global Synthesis Reveals Biodiversity Loss as a Major Driver of Ecosystem Change. *Nature.* 2012;486(7401):105–8. 10.1038/nature11118 22678289

[ref-2] Lewin HA Robinson GE Kress WJ: Earth BioGenome Project: Sequencing Life for the Future of Life. *Proc Natl Acad Sci U S A.* 2018;115(17):4325–4333. 10.1073/pnas.1720115115 29686065 PMC5924910

